# Loose Nanofiltration Membrane Incorporating CeZnFe Layered Double Hydroxide with Enhanced Dye/Salt Separation Performance and Self-Cleaning Ability

**DOI:** 10.3390/membranes13080711

**Published:** 2023-07-31

**Authors:** Cigdem Balcik, Bahar Ozbey-Unal, Busra Sahin, Ramazan Keyikoğlu, Alireza Khataee

**Affiliations:** 1 Department of Environmental Engineering, Gebze Technical University, 41400 Kocaeli, Turkey; bozbey@gtu.edu.tr (B.O.-U.); rkeyikoglu@gtu.edu.tr (R.K.); 2 Institute of Earth and Marine Sciences, Gebze Technical University, 41400 Kocaeli, Turkey; 3 Department of Biotechnology, Gebze Technical University, 41400 Kocaeli, Turkey; busrasahin@gtu.edu.tr; 4 Research Laboratory of Advanced Water and Wastewater Treatment Processes, Department of Applied Chemistry, Faculty of Chemistry, University of Tabriz, Tabriz 51666-16471, Iran

**Keywords:** loose nanofiltration, layered double hydroxides, dye/salt separation, self-cleaning

## Abstract

The high-salinity wastewater from the textile industry faces a significant challenge in effectively separating dyes and salts. In this study, a CeZnFe-layered double hydroxide (LDH)-incorporated nanofiltration (LNF) membrane was fabricated using the conventional interfacial polymerization (IP) technique to fractionate dyes and salts within the wastewater. The impact of CeZnFe LDH on various aspects of membrane performance was examined, including water flux, dye removal efficiency, dye/salt separation capability, self-cleaning ability, and membrane integrity. The addition of LDHs resulted in improved membrane surface hydrophilicity, thereby enhancing water flux. The optimized TFN membrane (0.050 wt% LDH in PIP solution) significantly improved pure water flux, exceeding 150%. All TFN membranes exhibited excellent performance in dye and salt fractionation (93% for Congo red, 2.6% for NaCl, and 40.7% for Na_2_SO_4_). Also, excellent self-cleaning ability was observed for the optimized membrane, exhibiting a remarkable water flux recovery rate after three operation cycles. Moreover, including CeZnFe LDH in the optimized TFN membrane played a significant role in enhancing membrane integrity. This study provides new inspiration for fabricating self-cleaning loose NF membranes using CeZnFe LDH for effective dye/salt separation.

## 1. Introduction

In recent years, many researchers have drawn attention to environmental pollution, the global food crisis, climate change, food security, and public health due to the adverse effects of increasing industrialization and the rapidly growing world population [1,2]. Many toxic substances and pollutants produced as waste in sectors such as textile and dye, food/feed, mining, paper mills, and pharmaceuticals significantly impact environmental pollution, the global climate crisis, public health, and food safety. In particular, textile and dye wastewater exhibit potential pollutants and toxic features in water sources due to their high chemical and organic content, pH, and salinity. When dye wastewater with high salinity mixes with water resources, it negatively affects the aquatic ecosystem and adversely affects agricultural irrigation and human health [2,3,4]. To manage this problem, conventional treatment techniques such as chemical oxidation, coagulation, and adsorption often produce toxic and pollutant by-products [2,5,6]. Therefore, treating high-saline and dye wastewater and recovering high-value-added compounds using membrane-based technologies are reported as important alternative techniques compared to conventional separation techniques [5,6].

There are several membrane-based separation technologies available for the treatment of different wastewater, including microfiltration (MF), ultrafiltration (UF), nanofiltration (NF), and reverse osmosis (RO). These membrane technologies are commonly utilized in wastewater treatment, desalination, pharmaceutical, and biotechnological applications [6]. Among these membranes, NF membranes are widely used in textile wastewater treatment for effective dye removal and dye/salt separation [7].

Typically, the structure of an NF membrane consists of a support layer made of UF membrane and a selective layer composed of an ultrathin polyamide (PA) layer [8]. NF membranes are classified based on the type of production material into polymeric membranes, ceramic membranes, or mixed matrix membranes. Polymer materials, such as selective permeability and pore sizes, strongly affect the membranes’ chemical composition and optimize their physical structures. Despite their high chlorine resistance, cellulose-based NF membranes are not preferred for NF applications owing to their low permeability performance. Polymer membranes incorporating nanoparticles are specifically engineered to enhance their transport properties and reduce membrane fouling [9]. The nanomaterials affect the porosity, permeability, pore size properties, and antifouling capacity of the membrane by modifying its chemical and physical structure. The presence of nanomaterials allows for better control and optimization of these membrane properties.

The commercial NF membranes are efficient for rejecting divalent cations and organic dyes but insufficient for separating dye/salts mixture [7]. Membrane fouling and high energy consumption, which cause lower water flux, are other challenges in NF-based membrane separation technologies [10,11]. The research focusing on the recovery of valuable compounds raises the need to develop new functional separation membranes characterized by high rejection for dyes and improved permeability behavior for salt [7].

Loose nanofiltration (LNF) membrane technologies have emerged as an economical, effective, and sustainable alternative for separating dye/salt mixtures while enabling salt recovery [5,7]. Similar to NF and UF membranes, LNF membranes have boundary pore sizes that effectively separate organic compounds (in the range of 500–2000 Da) from salt mixtures. Thus, LNF membranes can be used for the high permeability of salts through the membrane and to reject smaller organic compounds that cannot be retained in UF membranes [5,7]. Moreover, LNF membranes exhibit higher water flux performance at low pressure than NF membranes with tighter surface properties. The water channels present in the selective layer of the LNF membrane enable the passage of inorganic salts through the membrane while offering higher rejection of organic compounds. To reduce cross-linking in the selective layer and enhance the water flow permeability performance of LNF membranes, nanofillers such as nanoparticles, nanosheets, and nanofibers are incorporated into the polyamide layer [12]. This approach helps optimize the membrane structure and improve its overall performance. Zhu et al. fabricated a novel type of loose NF membrane (NFM) by incorporating GO-PSBMA (graphene oxide-poly(sulfobetaine methacrylate)) into a polyethersulfone (PES) matrix. This membrane exhibited impressive rejection rates, with 99.2% for Reactive Black 5 and 97.2% for Reactive Red 49, while showing a lower rejection rate of approximately 10% for bivalent salts for Na_2_SO_4_. Also, the membrane demonstrated good antifouling properties, as indicated by a high flux restoration of approximately 94.4% and a negligible decline in the total flux of around 0.18% [13]. In another study by Zhu et al. [14], a novel “loose” NF membrane was fabricated by blending Chitosan–Montmorillonite (CS–MMT) nanosheets using the phase inversion method. This membrane exhibited higher rejection rates for Reactive Black 5 and Reactive Red 49 while demonstrating lower rejection rates for bivalent salts than conventional NF membranes. Furthermore, the membrane’s antifouling performance increased the pure water flux from 32 to 68.82 Lm^−2^h^−1^.

Zhang et al. fabricated an antifouling LNF membrane. The dye and salt rejections of the membrane were reported as 99.2%, 98.6%, 75.8%, 4.4%, 15.6%, 17.7%, and 36.3% for methyl blue, Congo red, crystal violet, NaCl, MgCl_2_, MgSO_4_, and Na_2_SO_4_, respectively. The membrane also demonstrated a permeance of 42.9 Lm^−2^h^−1^bar^−1^. Moreover, it has been reported that AM-PEI/HPAN LNF membranes showed resistance to membrane fouling with a flux recovery rate of over 98% [15]. While there have been numerous studies on LNF membranes, further evaluation and research are still needed to enhance their water flux performance, antifouling capabilities, and photocatalytic properties [16,17]. Continued research efforts can lead to the development of more efficient and versatile LNF membranes with enhanced performance in these critical aspects.

Among all nanomaterials, LDHs are important alternative materials for modifying the water channels in the selective layer of LNF. LDHs offer various advantages, including their ion exchange capabilities, thermal stability, low production cost, biocompatibility, and compositional versatility [9,18,19]. Zhao et al. [20] fabricated an LDHs/polymer LNF membrane exhibiting impressive performance. The membrane achieved a rejection rate of 97.9% for methyl blue, 97.5% for acid fuchsin, and less than 3% for salt. The LDHs/polymer LNF membrane also demonstrated desirable antifouling activity and excellent hydrophilicity, with an 89.5% flux recovery ratio for humic acid. It is worth noting that there are limited studies available on LNF membranes incorporating LDHs, indicating the potential for further exploration and research in this area.

This study aims to report the fabrication of an LNF membrane by incorporating CeZnFe LDH using the conventional IP technique. The IP of piperazine (PIP) and 1, 3, 5-benzene tricarboxylic chloride (TMC) was conducted on a commercial substrate membrane (UP150, Microdyn Nadir, Wiesbaden, Germany) to fabricate a permeable-selective LNF membrane suitable for dye/salt separation. The effect of CeZnFe LDH on membrane performance was investigated in terms of water flux performance, antifouling properties, photocatalytic activity, dye removal efficiency, and the separation of dye/salt mixtures.

## 2. Materials and Methods

### 2.1. Materials

Polyether sulfone (PES) membrane (UP150, 150 kDa) was purchased from Microdyn Nadir GmbH (Wiesbaden, Germany). N-N-Dimethylacetamide (DMAc, 99%), n-hexane (96%), and piperazine (PIP, Mw = 86.14 g/mol) were purchased from Merck (Darmstadt, Germany). 1,3,5-benzene tricarboxylic chloride (TMC, 98%) and zinc chloride (ZnCl_2_, 99%) were obtained from Sigma-Aldrich (Darmstadt, Germany). Sodium chloride (NaCl, ≥99%), sodium sulfate (Na_2_SO_4_, ≥99%), sodium hydroxide pellets (NaOH, 99%), and Congo red (CR, Mw = 696.65 Da) were supplied from AFG Bioscience (Northbrook, IL, USA). Reactive Red 198 (RR, Mw = 984.21 g/mol) was purchased from Dystar (Raunheim, Germany). Iron(III) chloride hexahydrate (FeCl_3_6H_2_O) and cerium(III) nitrate hexahydrate (Ce(NO_3_)_3_.6H_2_O) were obtained from Sigma-Aldrich (Darmstadt, Germany).

### 2.2. Synthesis of CeZnFe LDH

CeZnFe LDH with a molar ratio of three between M^2+^ and M^3+^ species was synthesized through the co-precipitation method. Briefly, 3 mM ZnCl_2_, 0.5 mM FeCl_3_6H_2_O, and 0.5 mM Ce(NO_3_)_3_∙6H_2_O were dissolved in deionized water under a nitrogen (N_2_) atmosphere. The pH of the solution was adjusted to eight by gradually introducing NaOH while vigorously stirring the mixture. After a 24 h aging period, centrifugation was employed to separate the catalyst from the solution, and the resulting solid was subjected to multiple washes with ethanol and water. Subsequently, the solid was dried at 50 °C for 7 h to remove residual moisture. The CeZnFe LDH material was acquired by grinding and then sifting the resultant solid powder.

### 2.3. Fabrication of Membranes

**Figure 1:**
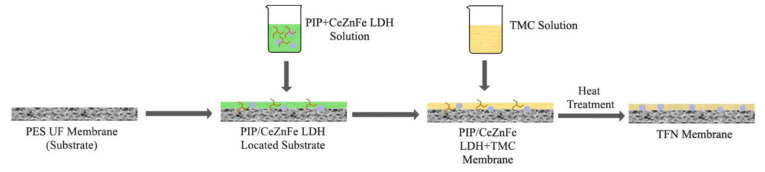
The scheme of fabrication of LNF membranes.

Thin film composite (TFC) and thin film nanocomposite (TFN) membranes were fabricated via conventional IP techniques using the commercial PES UF (UP 150, Microdyn Nadir) membrane as a substrate. The fabrication process involved depositing a polyamide layer onto the membrane substrate via interfacial polymerization between PIP and TMC monomers. PIP and various amounts of LDH were dispersed in deionized water to make a 0.8 wt% PIP solution containing different amounts of LDH. The fabrication procedure involved placing the substrate membrane in the module and pouring 20 mL of the PIP-LDH solution into the module, followed by a waiting period of five minutes at room temperature. After pouring out the excess solution, the membrane was detached from the module and allowed to undergo a drying process. Subsequently, 20 mL of TMC/n-hexane solution (0.1% (wt/v%)) was poured into the membrane after the dry membrane was placed in the module. The excess solution was discharged after a minute, and the membrane surface was rinsed with hexane to stop the polymerization between monomer and TMC/n-hexane. Lastly, the membrane was placed in an oven at 60 °C for 5 min to facilitate cross-linking. The fabricated membranes were subsequently immersed in deionized water. The fabricated membranes were denoted as M0, M1, M2, and M3, corresponding to LDH contents in the PIP solution of 0%, 0.025%, 0.050%, and 0.100%, respectively. The components of the fabricated membranes are provided in Table 1. Also, the scheme of fabricated LNF membranes is shown in Figure 1.

### 2.4. Characterization of CeZnFe LDH and Fabricated Membranes

The structural properties of the LDH were investigated using various analytical techniques. The crystalline phase was evaluated through X-ray diffraction using a PANalytical Empyrean instrument from the Netherlands. Fourier transform infrared (FTIR) analysis was performed using a Perkin Elmer Spectrum 100 instrument (Waltham, MA, USA). To examine the surface properties and elemental composition of the membrane, scanning electron microscopy (SEM) analyses were performed using a Philips XL30 SFEG (Eindhoven, The Netherlands) in conjunction with energy-dispersive X-ray spectroscopy (EDS). High-resolution transmission electron microscopy (HRTEM) was employed to capture TEM images, utilizing a Japan JEOL JEM-2100 Plus instrument operated at 200 kV. The Brunauer–Emmett–Teller (BET) method, implemented with the BELSORP model of Mini II from Japan, allowed for determining pore characteristics such as pore diameter, pore volume, and specific surface area. For X-ray photoelectron spectroscopy (XPS) analysis, a Thermo Scientific Escalab 250Xi+ instrument (Waltham, MA, USA) was utilized. Diffuse reflectance spectroscopy (DRS) measurements were carried out using an American Platinum PerkinElmer Lamda95 instrument (Waltham, MA, USA). The samples’ specific surface area, pore diameter, and pore volume were calculated using the BET method based on N2 adsorption–desorption isotherms obtained from the BELSORP model of Mini II.

The surface morphology and the elemental composition of the produced membranes were analyzed by SEM-EDS (Philips XL 30S FEG). An FTIR spectrum (Perkin Elmer FT-IR Spectrum 100) was used to determine the functional group composition of the membranes, and the wavenumber range was adjusted between 4000–650 cm^−1^. The static contact angle of the fabricated membranes was analyzed by contact angle measurements (KSV Attension T200 Theta).

### 2.5. Separation Performance of Fabricated Membrane

A dead-end filtration system (Sterlitech HP4750 Stirred Cell, Auburn, WA, USA) was utilized to evaluate the performance of the membranes. The effective membrane area was 14.6 cm^2^. The flux was calculated using Equation (1):

(1) $$J_{w}=\frac{M}{A\times t}$$

where J_w_ is the water flux (PWF), t is the filtration time, M is the permeate mass, and A is the effective area of the membrane.

The concentrations of dyes were determined using spectrophotometric analysis, while the concentrations of salts were measured through electrical conductivity measurement. The rejections (R) were calculated with Equation (2):

(2) $$R\left(\%\right)=\left(1-\frac{C_{p}}{C_{f}}\right)$$

where C_p_ represents the concentration of the permeate, and C_f_ corresponds to the concentration of the feed solution.

### 2.6. Photocatalytic Properties, Self-Cleaning Ability, and Membrane Integrity

The photocatalytic properties and self-cleaning ability of the fabricated membranes were investigated under a solar simulator integrated with a Xenon lamp (100 W) (Fytronix, Elazığ, Turkey). In the photodegradation procedure, the membranes were exposed to the dye solutions for a specific period until the permeate flux decreased. After filtration, the membrane surface was covered with dyes. To initiate the self-cleaning process, the membrane was first washed with distilled water to remove any unattached dyes from the membrane surface. The rinsed membrane was then immersed in a beaker containing deionized water. The beaker was positioned under the visible light source for 15 min. After the 15 min photodegradation period, the color change on the membrane surface was observed, and the pure water and dye solution fluxes were measured. This procedure was repeated three times. Furthermore, the potential disruption of membrane integrity by visible light exposure was assessed through SEM analysis, which allowed for the identification of any defects in the membrane morphology caused by visible light exposure.

## 3. Results and Discussion

### 3.1. Characterization of CeZnFe LDH

**Figure 2:**
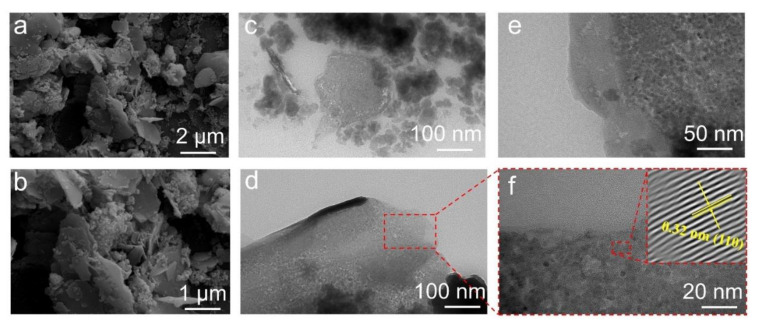
SEM images (**a**,**b**) and HRTEM images (**c**–**f**) of the CeZnFe LDH.

SEM analysis revealed the existence of uniformly dispersed two-dimensional particles exhibiting flake-like morphologies, which are specific to layered double hydroxides (LDHs) [21] (Figure 2a,b). In the TEM images, CeZnFe LDH appeared as distinct plate-like structures with well-defined edges and flat surfaces in a stacked arrangement. The high-resolution TEM images of LDHs provided further insight into the high crystal structure of LDHs and lattice fringes, from which the interplanar spacing of plane 110 was determined to be 0.32 nm (Figure 2c–f).

**Figure 3:**
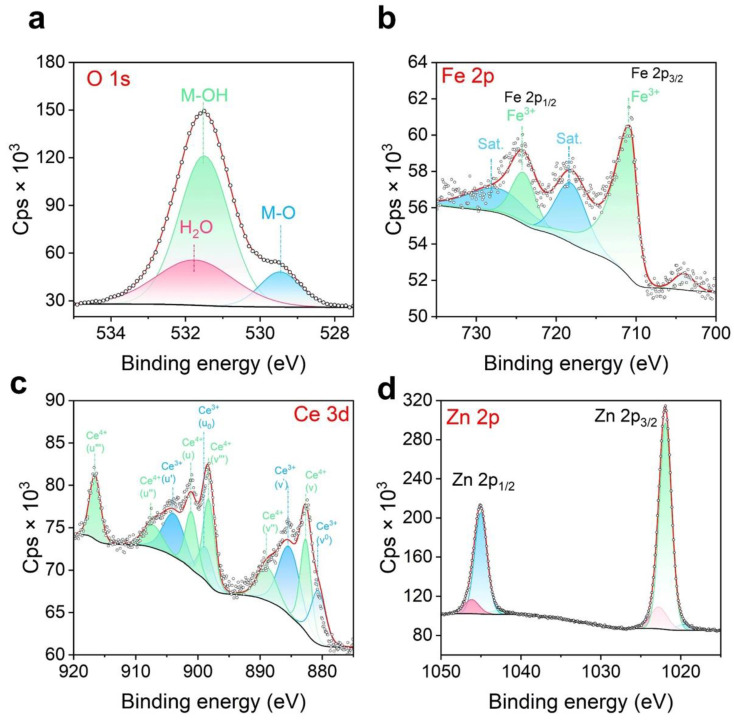
XPS images of the CeZnFe LDH: O 1s (**a**), Fe 2p (**b**), Ce 3d (**c**), Zn 2p (**d**).

XPS analysis was conducted to investigate the surface chemical composition of the CeZnFe LDH (Figure 3). The O1s signal was deconvoluted into three components having binding energies of 529.5 eV, 531.5 eV, and 531.8 eV, respectively. These components were attributed to different oxygen species: lattice oxygen (O_2_^2−^), chemisorbed and dissociated oxygen resulting from species such as adsorbed water molecules or adsorbed oxygen, and surface OH^−^ groups associated with metallic centers, respectively (Figure 3a) [22]. The Fe 2p XPS signal exhibited two peaks at 710.8 eV and 724.1 eV, accompanied by two satellite peaks at 718.1 eV and 727.1 eV (Figure 3b) [23]. The main peaks, separated by Δ13.3 eV, were attributed to the 2p3/2 and 2p1/2 spin states of Fe^3+^ in the CeZnFe LDH. A pre-peak with a binding energy of 703.9 eV, characterized by a single low-intensity peak, was also identified [24]. The Ce 3d spectrum of the Ce ZnFeLDH exhibited the presence of Ce^3+^ and Ce^4+^ in mixed valence states (Figure 3c) [25]. Deconvolution of the Ce 3d core-level XPS signal yielded 10 distinct components. Among these, the main peaks in the 3d_5/2_ region were assigned to components v_0_ (880.7 eV), v (882.6 eV), v′ (885.3 eV), v″ (888.8 eV), and v‴ (898.3 eV). The remaining five components in the 3d_3/2_ region were identified as u_0_ (898.7 eV), u (901.1 eV), u′ (903.9 eV), u″ (907.3 eV), and u‴ (916.6 eV) (Figure 3c). The Zn 2p_3/2_ and Zn 2p_1/2_ peaks represented different spin states of the Zn^2+^ ion peaks in the Zn 2p XPS spectrum (Figure 3d).

**Figure 4:**
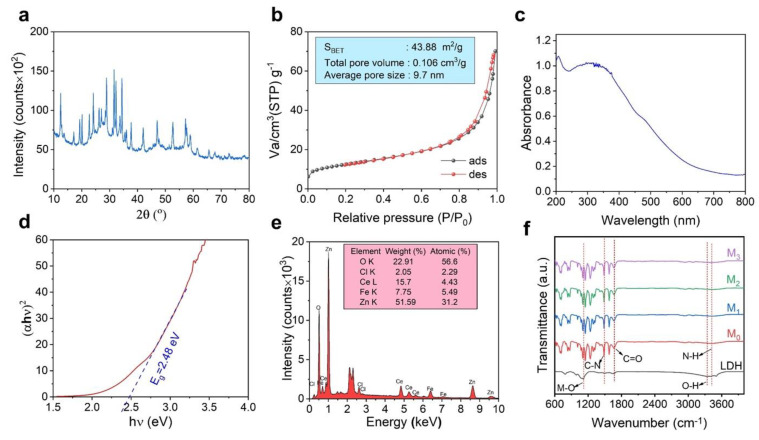
(**a**) XRD spectrum, (**b**) N_2_ adsorption–desorption isotherm, (**c**) UV–Vis diffuse reflectance spectrum, (**d**) bandgap evaluation, (**e**) EDS spectrum of the CeZnFe LDH, and (**f**) FTIR analysis of the CeZnFe LDH and fabricated membranes.

The XRD spectrum of CeZnFe LDH exhibited distinct diffraction peaks, indicating its well-defined crystal structure (Figure 4a) [26]. The diffraction peaks were observed at 2θ values of approximately 12.4°, 24.2°, 31.6°, 32,3°, 37.2°, 47.0°, 57.2°, and 59.0°, corresponding to the (003), (006), (110), (009), (015), (018), (110) and (113) crystallographic planes, respectively. The occurrence of these peaks suggests the formation of a well-ordered layered structure in the CeZnFe LDH material. The narrow peak widths indicate the presence of small-sized crystallites within the LDH material.

The N_2_ adsorption–desorption isotherm can be classified as an IV−type isotherm with a H_3_ hysteresis loop according to the IUPAC classification [27], indicating a material with slit-shaped pores belonging to packing plate-like particles [23] (Figure 4b). The material can be classified as mesoporous with an average pore diameter of 9.7 nm according to the Barrett–Joyner–Halenda (BJH) method using desorption data. The BET surface area and total pore volume of the CeZnFe LDH were found to be 43.88 m^2^/g and 0.106 cm^3^/g, respectively. The UV–Vis absorption spectrum of the CeZnFe LDH exhibited a maximum absorption peak in the UV region around 320 nm (Figure 4c). The bandgap energy (Eg) of the material was calculated according to the Kubelka–Munk formula, and the curve of (αhυ)^2^ against (hυ) is drawn as shown in Figure 3d, where the Eg value was 2.48 eV (Figure 4d) [24].

The elemental composition of the CeZnFe LDH was determined using an energy-dispersive X-ray spectroscopy (EDX) analysis (Figure 4e). The major elements detected were Zn, Fe, Ce, O, and Cl, which are the constituents of the LDH structure. Quantitative analysis of the atomic percentages of each element in the CeZnFe LDH material indicated that the molar ratio of Zn:(Fe+Ce) was very close to the precursor amounts during synthesis, implying the successful incorporation of these elements within the LDH structure.

### 3.2. Characterization of Fabricated Membranes

The FTIR spectrum of CeZnFe LDH exhibited several characteristic absorption bands (Figure 4f). The broad absorption band occurred in the range of 3400–3600 cm^−1^, indicating the existence of hydroxyl (OH) stretching vibrations. This suggests the presence of interlayer water molecules and hydroxyl groups associated with the LDH structure [24]. Moreover, the peaks at approximately 1380 cm^−1^ and 1100 cm^−1^ were assigned to the asymmetric and symmetric stretching vibrations of the metal–oxygen (M–O) bonds, respectively [28]. These peaks indicate the presence of metal–oxygen bonds within the LDH structure. Observing absorption bands in the region below 1000 cm^−1^ was associated with the vibrations of metal–oxygen–metal (M–O–M) bonds, confirming the presence of the layered structure in the LDH material.

**Figure 5:**
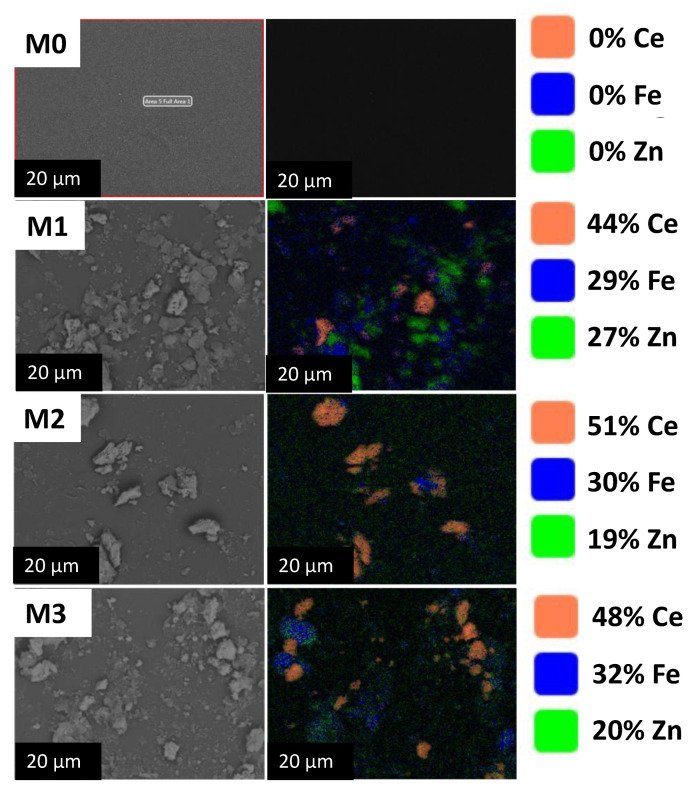
SEM images and EDS mappings of the fabricated membranes with different Ce ZnFe loadings.

The morphological properties of the LNF membranes incorporating Ce ZnFe LDH nanomaterials are shown in Figure 5. Figure 5 indicates that the pristine LNF membrane (M0) surface appeared smooth. However, upon the addition of CeZnFe LDH, the surface of the membrane became rough, indicating the presence of nanomaterial. The lamellar structures of the CeZnFe LDH were observed on the membrane surface, which is characteristic of the LDH [19]. Furthermore, the elemental composition of the fabricated membranes was determined using EDS mapping. The EDS spectra of the modified membranes confirmed the successful deposition of Ce, Fe, and Zn elements on the membrane’s top surface. This analysis provides evidence of CeZnFe LDH incorporation within the LNF membrane structure.

**Figure 6:**
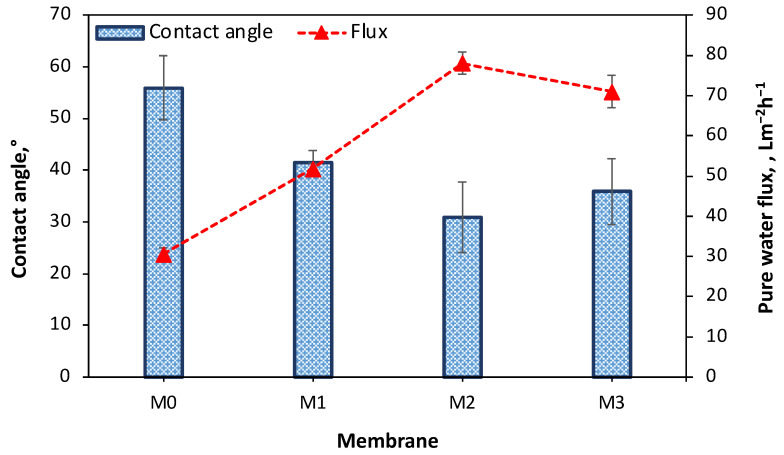
CA and PWF of the TFC and TFN membranes.

The water contact angle (CA) values of the TFC and TFN membranes were determined, and the results are presented in Figure 6. The CA values for M0, M1, M2, and M3 membranes were 55.9°, 41.5°, 30.9°, and 35.9°, respectively. Figure 6 shows that incorporating CeZnFe LDH on the TFN membranes decreased the water contact angle. This decrease can be attributed to the hydrophilic nature of the LDH, which promotes a greater affinity for water [29]. Also, the Wenzel state of the rough surfaces can explain the decrease in water contact angle according to the extended Young’s model. The presence of rough surface structures increases the effective surface area of the solid surface. Thus, increased roughness results in improved hydrophilicity in the case of a hydrophilic membrane surface structure [30,31]. The presence of intercalated hydroxyl functional groups within the structure of CeZnFe LDH can be attributed to their hydrophilic characteristic. This hydrophilicity plays a crucial role in the performance of the LNF membranes. When the LDH content in the PIP solution increases, the pure water flux of the membranes shows a significant improvement, increasing by more than 150%. This means that the membranes incorporating CeZnFe LDH exhibit a greater affinity for water, allowing for better solubility and transport of water molecules through the membrane structure [32,33]. In addition to the hydrophilic nature of CeZnFe LDH, the presence of interlayer voids within these nanoparticles can enhance water flux. These interlayer voids provide additional free volume in the flow channels, allowing better water transport through the membrane. However, it is important to note that in the case of the M3 membrane, there was a deterioration in pure water flux. This can be attributed to the aggregation of CeZnFe particles on the membrane’s top surface. When nanoparticles agglomerate, they can increase the membrane’s tortuosity, which hinders water transport through the membrane [34]. Therefore, while the interlayer voids in CeZnFe nanoparticles can enhance water flux, it is crucial to maintain uniform dispersion and prevent agglomeration by optimizing the amount of LDH content in the PIP solution.

### 3.3. Dye/Salt Separation Performance of Fabricated Membranes

**Figure 7:**
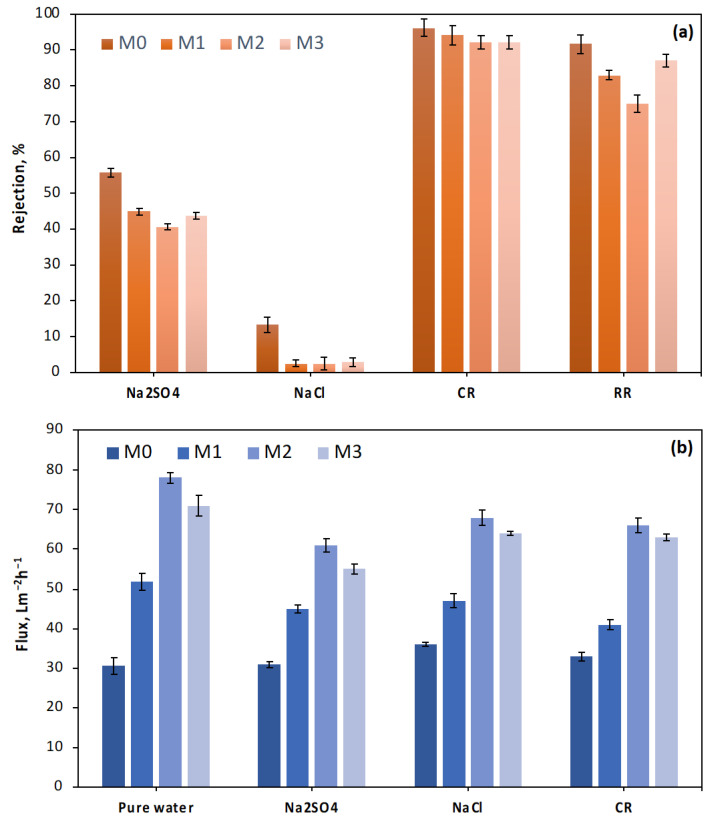
(**a**) Dye and salt rejection ratios and (**b**) flux values of the fabricated TFC and TFN membranes.

The fabricated membranes’ dye/salt separation filtration performance was evaluated using Na_2_SO_4_, NaCl, acid dye (CR), and reactive dye (RR) solutions. The rejection performances and the permeate water fluxes of the TFC and TFN membranes with various LDH loadings are shown in Figure 7. Higher rejection rates for the dye molecules and lower rejection rates for the salt ions are desirable for effective dye/salt separation. The rejection performance of the loose TFN membranes for ions can be attributed to two important mechanisms: sieving and dielectric exclusion effects. Smaller ions can easily pass through these pores according to the sieving effect, while larger ions are hindered or completely blocked. The dielectric exclusion effect relies on the electrical properties of the membrane and the ions [20]. Including CeZnFe LDH content in TFN membranes results in a positive surface charge, enhancing the electrostatic repulsion for divalent ions compared to monovalent ions. As a result, the rejection of Na_2_SO_4_ salts is higher compared to NaCl salts, and the transmittance of inorganic salts is higher for TFN membranes compared to TFC membranes (less than 45% for Na_2_SO_4_ and 3% for NaCl). All the TFC and TFN membranes exhibited high separation performance for CR and RR dyes. It was observed that the amount of CeZnFe LDH content in TFN membranes did not have a notable impact on the separation performance for all types of wastewater. The permeate water fluxes significantly improved as the CeZnFe LDH content in the PIP solution increased. In particular, when the CeZnFe LDH content in the PIP solution was raised to 0.050 wt%, the permeate water fluxes increased by more than almost 100% for all solutions, owing to the improved hydrophilicity of the TFN membranes.

Additionally, flow channels in LDH nanoparticles may further enhance the water flux [29]. Although the M2 membrane exhibited the highest permeate water flux, there was an insignificant reduction in rejection for all types of wastewater due to the trade-off effect [35]. The permeate flux of the M3 membrane slightly decreased for all types of solutions, which aligns with the contact angle analysis of the M3 membrane, highlighting the importance of the amount of added CeZnFe LDH content. As a result, the TFN membranes demonstrated satisfactory performance in dye and salt filtration, showcasing their ability to separate and recover valuable inorganic salts and dyes from wastewater. Based on these results, the CeZnFe LDH concentration in the PIP solutions was fixed at 0.050 wt% for the following experiments.

The mean pore radius was calculated with the Guerout–Elford–Ferry equation. The approximate pore size of the membranes was calculated as 5.1 nm (M0) and 6.3 nm (M2) according to the equation [9]. This slight increase in pore size did not significantly affect the rejection ratio of the dye molecule. It was reported that dye molecules have a tendency to cluster together in aqueous solutions due to intermolecular hydrogen bonds and/or hydrophobic interactions. As a result, the actual size of the dye in the aqueous solution is larger than that of a single dye molecule [6]. Therefore, the membranes can exhibit significant dye rejection even if their MWCO is higher than the MWCO of the dye.

The dye adsorption capacity of the membranes can impact their separation performance. Hence, the dye adsorption capacity of both M0 and M2 membranes was evaluated with a static adsorption experiment [6]. The test revealed that M0 and M2 membranes showed adsorption amounts of 3.21 mg/g and 3.42 mg/g, respectively. Although incorporating LDH slightly enhances the dye adsorption of the membranes, the increase is not substantial enough to significantly affect their dye rejection capabilities. Instead, the dominant separation mechanism of the membrane is attributed to the synergistic effects of size exclusion and the Donnan effect.

### 3.4. Self-Cleaning Ability and Membrane Integrity

**Figure 8:**
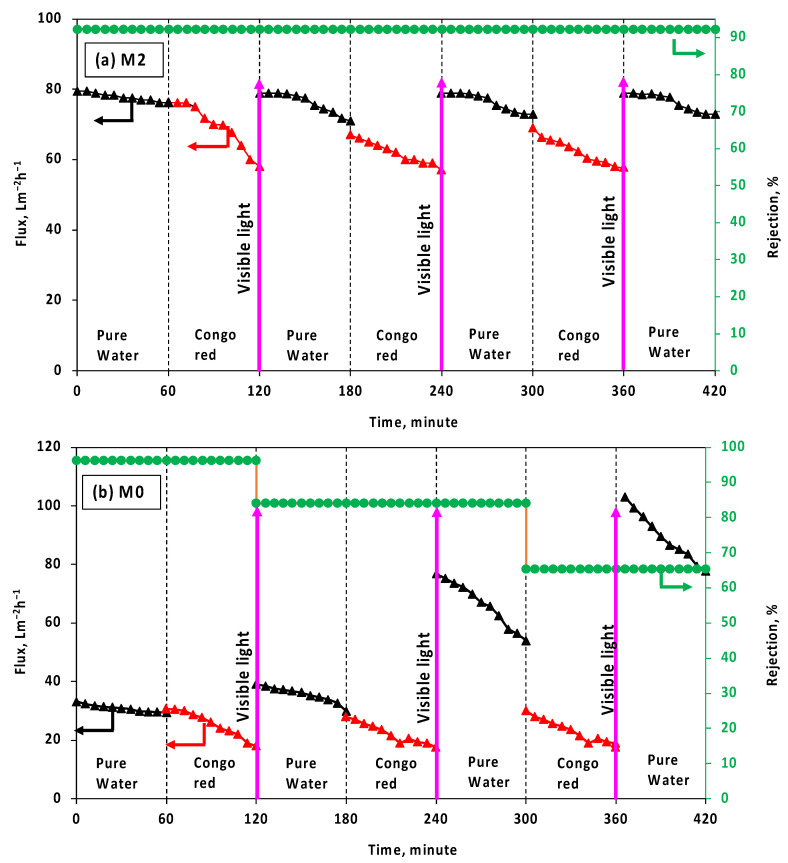
The water flux and rejection performances of the selected (**a**) M2 and (**b**) M0 membranes before and after the photocatalytic process.

**Figure 9:**
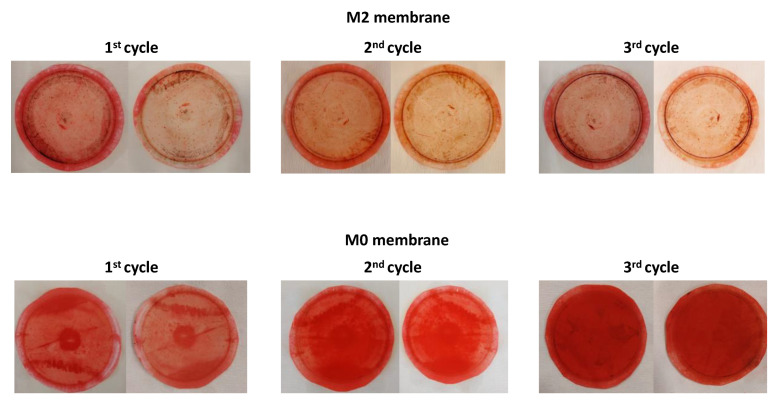
The photographs of the selected M0 and M2 membranes before and after the photocatalytic process for each cycle.

The self-cleaning ability and membrane integrity of the TFC and selected TFN membranes were examined using Congo red dye. The membranes’ permeate water flux and CR rejection performances were assessed before and after visible light photocatalytic degradation, as depicted in Figure 8. In the first cycle, the water flux of the M2 membrane decreased from 76.2 Lm^−2^h^−1^ to 58 Lm^−2^h^−1^ for the CR solution. However, after a 15-min photodegradation of the dye under visible light, the water flux recovered to approximately 78.9 Lm^−2^h^−1^ for pure water, demonstrating the outstanding self-cleaning ability of the M2 membrane. The water flux was restored to its initial pure water flux level (approximately 79.5 Lm^−2^h^−1^) after a 15-min photocatalytic process in the second and third cycles. However, the initial water flux for the CR solution slightly decreased after each cycle. For all cycles, the M2 membrane exhibited good stability during the filtration and photocatalytic processes, maintaining a high rejection rate for CR removal. In the first cycle filtration test of the CR solution, the initial water flux of the M0 membrane decreased from 30.7 Lm^−2^h^−1^ to 18 Lm^−2^h^−1^. This reduction in water flux indicates fouling on the membrane surface due to the presence of CR dye. Surprisingly, it was observed that the pure water flux of the M0 membrane tended to increase after each cycle of the photocatalytic process. However, the rejection rate decreased below 70% in the third cycle, indicating a possible negative impact of photocatalytic degradation on the characteristics of the M0 membrane. This decline suggests that the photocatalytic degradation process might have a negative impact on the characteristics of the M0 membrane. The reduction in rejection rate indicates that the membrane’s ability to reject CR decreased, potentially due to changes in its surface properties or structure. It was attributed that PES is a photo-unstable polymer containing a chromophore group known as the phenoxy-phenyl sulfone group, which is abundant in (bonds that can interact with a light source and undergo degradation [36]. The photographs of the M0 and M2 membranes before and after each cycle of the photocatalytic process are presented in Figure 9. It is evident from the images that the presence of dye contaminants on the M2 membrane surface was significantly reduced after the photocatalytic process, providing strong evidence of the excellent self-cleaning ability of the M2 membrane, as also supported by Figure 8. In contrast, it was observed that the density of dye contaminants on the M0 membrane surface increased even with the application of visible light after each cycle. This observation indicates that adding CeZnFe LDH to the PIP solution enhances the membrane’s self-cleaning ability. The loss of particles was also studied with a long-term filtration process. After filtration, both feed and permeate streams were analyzed with ICP-OES to measure Zn, Fe, and Ce. Zn, Fe, and Ce were not detected in the permeate stream. In the case of the concentrate stream, while the concentrations of Fe were measured at 0.044 mg/L, Zn and Ce were not detected. As can be seen from the results, no significant particle loss was observed during long-term filtration for the M2 membrane.

**Figure 10:**
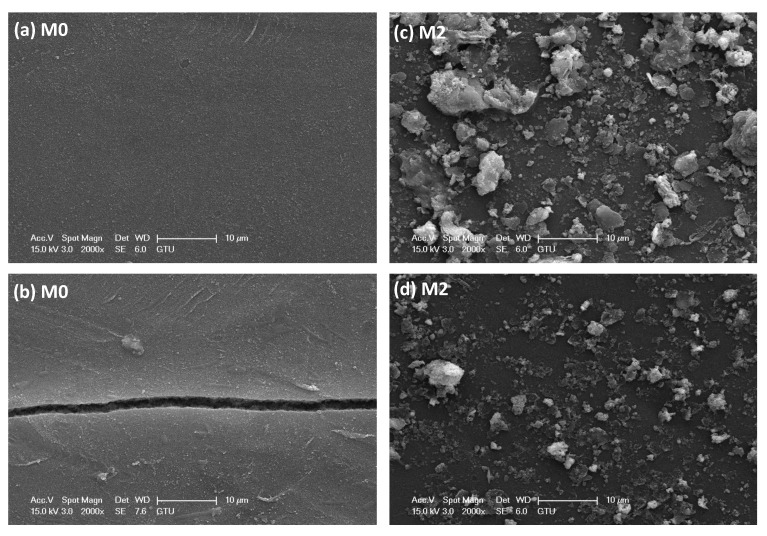
SEM images of the (**a**,**b**) M0 and (**c**,**d**) M2 membranes before and after 45 min photocatalytic process.

The effect of visible light on membrane integrity was investigated through SEM analysis (Figure 10). The SEM images in Figure 10 revealed the presence of a crack on the M0 membrane surface, which explains the increased pure water flux and worsening rejection after the photocatalytic process. This phenomenon can be attributed to the absorption of energy on the membrane surface, leading to the generation of a significant quantity of free radicals and subsequent surface deformation of the membrane [37]. In contrast, it was observed that the dye contaminants on the M2 membrane surface decreased after the photocatalytic process, with no surface deformation observed. Due to the visible light exposure, the LDH electrons are stimulated and combined with nearby H_2_O and O_2_ to generate hydroxyl active radicals (^•^OH). These radicals facilitate the oxidation of organic dyes, breaking them down into smaller molecules such as H_2_O and CO_2_, eliminating the dye from the membrane surface [6].

## 4. Conclusions

In summary, a loose NF membrane was successfully developed using CeZnFe LDH in a PIP solution through the conventional IP procedure. The performance of TFC and TFN membranes was evaluated in terms of dye/salt separation, self-cleaning ability, and membrane integrity. The integration of CeZnFe LDH (0.050 wt% in PIP solution) in the M2 membrane led to a substantial improvement in pure water flux, exceeding 150%, attributed to improved hydrophilic characteristics and the formation of free-flow channels within the CeZnFe LDH structure. The rejection of inorganic salts was higher for TFC membranes compared to TFN membranes. In contrast, all the membranes demonstrated excellent separation performance for CR and RR dyes, indicating the TFN membranes’ ability to effectively separate and recover valuable inorganic salts and dyes. Furthermore, the M2 membrane has excellent self-cleaning ability and exhibits a remarkable water flux recovery rate after three operation cycles. Additionally, the incorporation of CeZnFe LDH in the M2 membrane contributed to improved membrane integrity by acting as an effective barrier, shielding polymeric membranes from photocatalytic degradation. This study provides new inspiration for fabricating self-cleaning loose NF membranes using CeZnFe LDH for effective dye/salt separation.

## Figures and Tables

**Table 1:** Components of the membrane casting solution.

Membrane Type	PIP (wt%)	TMC/n-Hexane (wt/v%)	LDH Content in PIP (wt%)
M0	0.8	0.10	0.000
M1	0.8	0.10	0.025
M2	0.8	0.10	0.050
M3	0.8	0.10	0.100

## Data Availability

The data presented in this study are available in the article.
